# Cardio-oncology in advanced prostate cancer

**DOI:** 10.3389/fonc.2024.1386597

**Published:** 2024-06-14

**Authors:** Kenneth Chen, Ting Hong Wong, Yu Guang Tan, Kae Jack Tay, Wei Chong Tan, Johan Chan, Henry Ho, Christopher Cheng, Jeremy Yuen-Chun Teoh, Peter Ka-Fung Chiu, Hung Jen Wang, Marniza Binti Saad, Ravindran Kanesvaran, You Quan Li, Choon Ta Ng, Jeffrey Kit Loong Tuan, John Shyi Peng Yuen

**Affiliations:** ^1^ Department of Urology, Singapore General Hospital, Singapore, Singapore; ^2^ Division of Surgery and Surgical Oncology, National Cancer Centre Singapore, Singapore, Singapore; ^3^ Yong Loo Lin School of Medicine, National University of Singapore, Singapore, Singapore; ^4^ Division of Medical Oncology, National Cancer Centre Singapore, Singapore, Singapore; ^5^ S. H. Ho Urology Centre, Department of Surgery, Faculty of Medicine, The Chinese University of Hong Kong, Hong Kong, Hong Kong SAR, China; ^6^ Department of Urology, Kaohsiung Chang Gung Memorial Hospital and Chang Gung University and College of Medicine, Kaohsiung, Taiwan; ^7^ Department of Clinical Oncology, University of Malaya Medical Centre, Kuala Lumpur, Malaysia; ^8^ Division of Radiation Oncology, National Cancer Centre Singapore, Singapore, Singapore; ^9^ Department of Cardiology, National Heart Centre Singapore, Singapore, Singapore

**Keywords:** androgen deprivation therapy, advanced prostate cancer, cardio-oncology, cardiovascular health, androgen receptor pathway inhibitors, abiraterone, enzalutamide, metastatic prostate cancer

## Abstract

Treatment intensification with androgen deprivation therapy (ADT) and androgen receptor pathway inhibitors (ARPi) have led to improved survival in advanced prostate cancer. However, ADT is linked to significant cardiovascular toxicity, and ARPi also negatively impacts cardiovascular health. Together with a higher prevalence of baseline cardiovascular risk factors reported among prostate cancer survivors at diagnosis, there is a pressing need to prioritise and optimise cardiovascular health in this population. Firstly, While no dedicated cardiovascular toxicity risk calculators are available, other tools such as SCORE2 can be used for baseline cardiovascular risk assessment. Next, selected patients on combination therapy may benefit from de-escalation of ADT to minimise its toxicities while maintaining cancer control. These patients can be characterised by an exceptional PSA response to hormonal treatment, favourable disease characteristics and competing comorbidities that warrant a less aggressive treatment regime. In addition, emerging molecular and genomic biomarkers hold the potential to identify patients who are suited for a de-escalated treatment approach either with ADT or with ARPi. One such biomarker is AR-V7 splice variant that predicts resistance to ARPi. Lastly, optimization of modifiable cardiovascular risk factors for patients through a coherent framework (ABCDE) and exercise therapy is equally important. This article aims to comprehensively review the cardiovascular impact of hormonal manipulation in metastatic hormone-sensitive prostate cancer, propose overarching strategies to mitigate cardiovascular toxicity associated with hormonal treatment, and, most importantly, raise awareness about the detrimental cardiovascular effects inherent in our current management strategies involving hormonal agents.

## 1 Introduction

The success of hormonal therapy in suppressing testosterone levels in men with advanced prostate cancer was first reported by Huggins and Hodges in 1941 (1, 2). With the subsequent widespread adoption of androgen deprivation therapy (ADT) through surgical and medical castration techniques, ADT has been established as the fundamental tenet of treatment in advanced prostate cancer. This may include patients diagnosed with high-risk prostate cancer, *de novo* metastatic disease, or recurrence of prostate cancer.

Furthermore, landmark trials of combination therapy for metastatic hormone-sensitive prostate cancer (mHSPC) have all included the use of ADT in the intervention arms, as have ongoing trials of novel combination therapeutics, thus consolidating its utility in every phase of the disease (3). Meanwhile, survival of patients with advanced prostate cancer is also increasing due to improved therapeutic agents, with Corsini and colleagues (4) recently reporting a median overall survival of four to five years. As a result, patients’ lifetime exposure to ADT is expected to rise in tandem. This increasing runway of ADT treatment leads to new challenges and a different set of systemic adverse effects associated with castration that advanced prostate cancer patients will face in their treatment journey.

## 2 Cardiometabolic impact of hormonal treatment

The cardiometabolic effects of ADT and androgen receptor pathway inhibitors (ARPi), specifically abiraterone and enzalutamide (5) have been well-reported in the literature. In a large SEER-based study including 140 474 patients diagnosed with non-metastatic prostate cancer, the administration of GnRH agonists was associated with a higher risk of CAD, AMI, and SCD compared to ADT naive patients (26.9%, 16.6%, 17.7% *vs* 25.1%, 14.8%, 14.2%, respectively) (6). In a large epidemiological study based on 6556 and 3330 Swedish men who received GnRH agonists and orchiectomy as primary treatment respectively, the 10-yr crude probability of cardiovascular disease was 0.56 (95% CI 0.55–0.57) and 0.52 (95% CI 0.50–0.54) respectively (7). Meta-analyses have revealed a 1.4 times higher likelihood of cardiac events (incidence ~14%) including ischemic heart disease, myocardial infarction, tachyarrhythmia, and heart failure associated with abiraterone but not with enzalutamide, even though both significantly doubles the risk of hypertension (overall incidence of around 20%) (8, 9). Narayan and colleagues (10) show that hormonal treatment may predispose patients to multifarious cardiometabolic disease and proposes a systematic approach that includes monitoring and addressing modifiable CV risk factors and a model of multidisciplinary care.

Aside from this, Kaur and Werstruck (5) suggest that testosterone may have a wide array of beneficial physiological effects. Adequate testosterone levels in men shorten the heart-rate-corrected QT interval (QTc) (11), and induce vasodilation by downregulation of L-type voltage-gated calcium channels (12) and upregulation of calcium-activated potassium channels (13). Additionally, testosterone improves cardiac contractility (12) and cardiomyocyte relaxation speed (14). It also strengthens glycaemic control by improving insulin sensitivity, HbA1c levels, and fasting blood glucose levels (15). Furthermore, testosterone has possible effects on the reduction of atherosclerosis by slowing atheroma progression and reversing lipid deposition on arterial walls (16). In particular, numerous trials (17, 18) have shown an association between testosterone replacement therapy and reduced total and low-density lipoprotein cholesterol levels. Testosterone also inhibits the differentiation of pluripotent stem cells into adipocytes (19, 20), and may prevent centripetal obesity through stimulation of lipolysis (21). It may also reduce systemic inflammation via unclear effects on high-sensitivity C-reactive protein and inflammatory cytokines (22), though this remains controversial (23).

Based on these findings, the suppression of testosterone levels by ADT may predispose patients to numerous adverse outcomes. First, ADT may lead to QTc prolongation (11), thus increasing the risk of ventricular arrhythmias and sudden cardiac death (24). Gonadotropin-releasing hormone (GnRH) agonists may also increase cardiovascular risk (25) by causing a reduction in vasodilation (12, 13), thus leading to chronic hypertension, as well as diminished cardiac contractility and cardiomyocyte relaxation (14). Third, it may increase arterial stiffness and accelerate the development of atherosclerosis (26); as a further consequence, ADT may induce the activation of naive T cells expressing GnRH receptors, causing clonal expansion and differentiation into Th1 cells, which in turn activate macrophages; the macrophages release matrix-degrading proteases which degrade the thin connective tissue cap of atherosclerotic plaque, resulting in plaque rupture (27). Fourth, ADT may accelerate the development of diabetes (HR: 1.44; 95% CI: 1.34–1.55) (28) by increasing insulin resistance (29) and precipitating a pro-inflammatory milieu (22), with Inaba and colleagues (30) reporting an association between ADT and hyperglycaemia as well as impaired pancreatic beta cell function. Lastly, patients on ADT are predisposed to obesity due to the loss of androgen-mediated inhibition of stem cell differentiation into adipocytes (19, 20) and reduction of androgen-induced lipolysis (21). There is a strong body of evidence illustrating how obesity directly increases the likelihood of developing cardiovascular risk factors such as dyslipidaemia, type 2 diabetes mellitus, hypertension, and sleep disorders. Additionally, obesity independently contributes to the onset of cardiovascular disease and mortality, separate from its impact on other cardiovascular risk factors (31).

## 3 The heart of the matter

Indeed, numerous studies have shown an association between ADT treatment for prostate cancer and increased adverse cardiovascular events. In a pooled analysis of three large randomised trials, D’Amico and colleagues (32) showed that the use of ADT treatment for six months was associated with earlier onset of fatal myocardial infarctions in patients aged 65 years or older. Furthermore, a large analysis of Surveillance, Epidemiology and End Results Medicare data (28) showed that GnRH agonist treatment for men with locoregional prostate cancer was associated with higher risk of coronary heart disease (adjusted hazard ratio [HR]: 1.16, p < 0.001), myocardial infarction (adjusted HR: 1.11, p = 0.03), and sudden cardiac death (adjusted HR: 1.16, p = 0.004).

The importance of cardiovascular outcomes when considering the adverse effects of ADT is further highlighted by the significant prevalence of cardiovascular risk factors (CVRFs) in men with prostate cancer. In a recent analysis (33), nearly all patients studied (99%) had at least one uncontrolled modifiable CVRF, and more than half (51%) had three or more uncontrolled modifiable CVRFs. In addition, poor control of CVRFs was also shown to occur regardless of ADT use or a history of cardiovascular disease. Similarly, up to 90% of men receiving ADT in the HERO trial by Shore and colleagues (34) had CVRFs or frank cardiovascular disease at baseline.

Ultimately, it should come as little surprise that the leading cause of death in patients with prostate cancer is not the cancer itself or the effects thereof, but rather the development of cardiovascular disease. Sturgeon et al, in a large SEER based population study of cardiovascular disease mortality risk in US cancer patients, showed that in 509128 patients diagnosed and treated for prostate cancer, 17.6% of deaths were cancer-related and 16.6% were cardiovascular-related deaths of which an overwhelming 77.3% were heart disease-related. More importantly, the historical trends from the study showed that since the 1990s, cardiovascular-related deaths have overtaken cancer-related deaths for prostate cancer patients (35).

As such, given the increasing lifetime exposure of patients to ADT, and the considerable effects of ADT on cardiovascular morbidity and mortality, it is prudent to establish strategies to optimize cardiovascular outcomes in men with advanced prostate cancer, to improve their overall survival and quality of life.

## 4 Strategies to optimize cardiovascular outcomes in advanced prostate cancer

**Figure 1:**
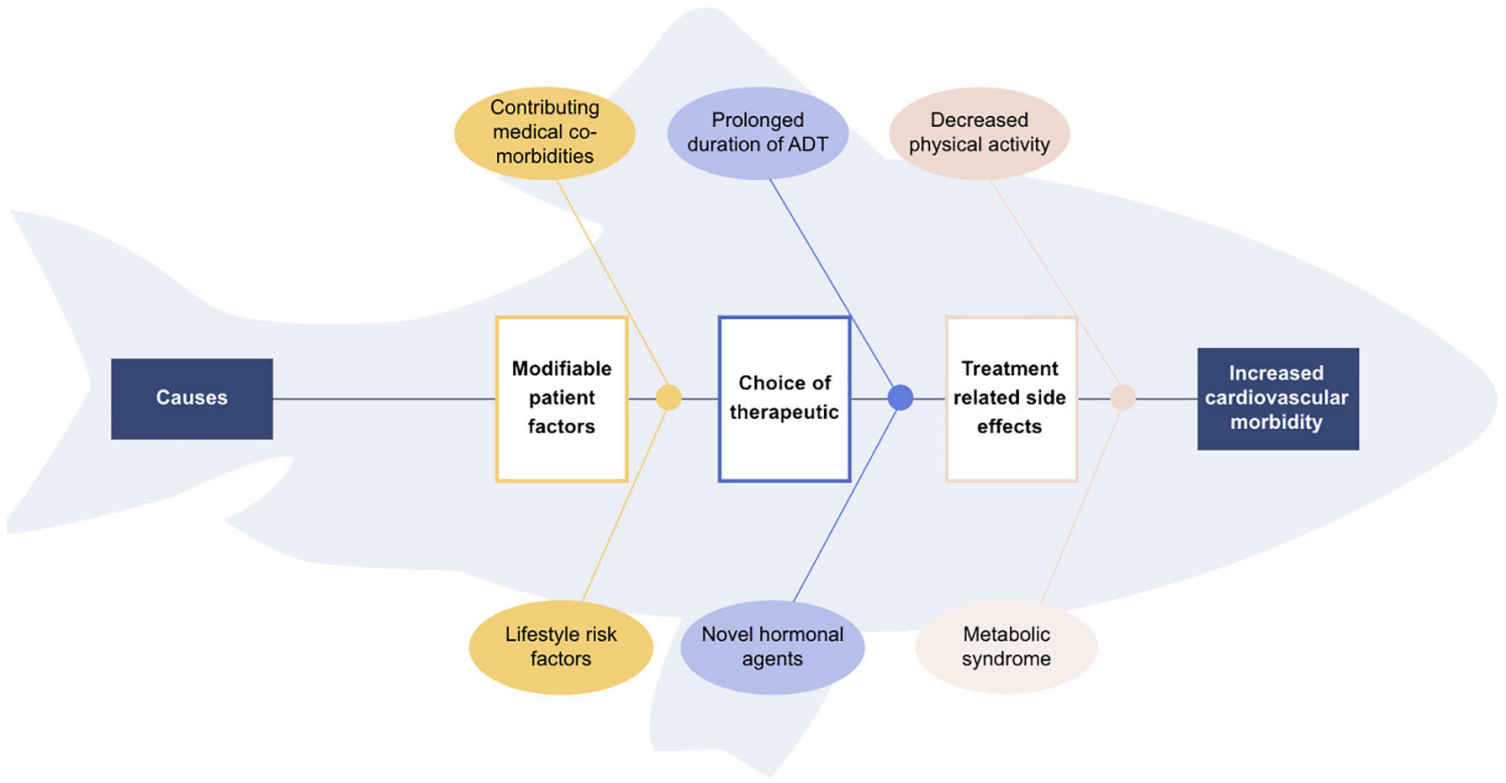
Fishbone diagram of contributing causes for increased cardiovascular morbidity in metastatic prostate cancer patients.

Adopting a fishbone analysis of the negatively impacted cardiovascular health of advanced prostate cancer patients on hormonal treatment (**Figure 1**), we can identify various contributing factors and thus propose four strategies to mitigate the long-term adverse cardiovascular effects: the use of pharmacological alternatives, de-escalation of ADT, provision of cardiovascular risk assessment and care, and optimizing patient factors with a focus on exercise.

### 4.1 Use of pharmacological alternatives

First, the use of GnRH antagonists such as Degarelix and Relugolix can be considered for ADT, rather than GnRH agonists. A pooled analysis (36) of six Phase III randomized trials comparing GnRH antagonists and agonists showed that Degarelix reduced cardiovascular events among patients with prior cardiovascular disease (HR: 0.44, 95% CI: 0.26 - 0.74, p = 0.002). Absolute risk reduction during the first year of treatment was 8.2%, yielding a number needed to treat of 12. As for Relugolix, an oral luteinizing hormone–releasing hormone (LHRH) antagonist, Shore and colleagues (34) showed a significant 54% reduction in the risk of major cardiovascular events compared to the GnRH agonist, Leuprolide. The effect size was comparable to the abovementioned study of Degarelix (HR: 0.46; 95% CI:0.24–0.88), though Relugolix may bring a larger benefit to patients with prior cardiac disease (HR 0.20 *vs* 0.46) (34). However, the data is not all that homogenous. Lopez et al. reported on the PRONOUNCE trial - the first, international, randomized clinical trial comparing the cardiovascular safety of GnRH antagonists with GnRH agonists in patients with prostate cancer. Men with confirmed prostate cancer and a history of ASCVD planned for ADT were randomized 1:1 to receive either a starting dose of degarelix 240mg followed by 11 maintenance doses or leuprolide 22.5mg intramuscular injection followed by 3 similar injections every 3 monthly. The trial was terminated prematurely due to slow recruitment and fewer than projected number of major adverse cardiovascular event (MACE) events, defined as a composite of all-cause death, myocardial infarction, or stroke through 12 months. No significant difference in MACE was demonstrated between the 2 groups (hazard ratio, 1.28 [95% CI, 0.59–2.79]; P=0.53) due to multiple limitations of the trial, not least the fact that it was open-label and active follow-up with a cardiologist was mandatory for all patients. These factors led to unrealistic and biased cardiac care in the study population (37). Nonetheless, a recent systematic review that included the data from PRONOUNCE showed that compared with GnRH agonists, the pooled OR of GnRH antagonists for MACE was 0.57 (95% credible interval: 0.37–0.86) and 0.58 (95% credible interval: 0.32–1.08) for all-cause death, supporting a cardio-protective effect for GnRH antagonists (38).

Second, the paradigm shift of the standard of care to combination therapy for *de novo* mHSPC has cast attention on ARPi and their effects on cardiovascular health as well. While ARPi therapy has demonstrated dramatic attenuation of testosterone signalling and oestrogen levels, with effects even more profound than that of traditional ADT, treatment with ARPi has also been shown to compound cardiovascular risk in men with prostate cancer (8). Two meta-analyses in the literature have compared the cardiovascular toxicity of abiraterone and enzalutamide. Moreira and colleagues (8) found that abiraterone use in conjunction with prednisone was associated with a statistically significant 76% increase in the risk of high-grade cardiac disorder adverse events (RR: 1.76, CI 95: 1.12–2.75, p = 0.01) and 28% increase in all-grade cardiac disorder adverse events (RR: 1.28, CI 95: 1.06–1.55, p = 0.01), while enzalutamide was not associated with an increase in the risk of all-grade or high-grade cardiac disorder adverse events. Meanwhile, Iacovelli and colleagues (9) reported that abiraterone was found to significantly increase the risk of both cardiac toxicity and hypertension, whereas enzalutamide only significantly increased the risk of hypertension.

Overall, the results of both meta-analyses suggest that abiraterone is associated with a much more significant increase in cardiovascular toxicity than enzalutamide. Consequently, this may inform our choice in patients with significant CVRFs and for whom cardio-oncology is a priority in management goals. A more recent meta-analysis confirmed the increased risks of next-generation androgen receptor-targeted agents on cardiac-related adverse events, hypertension, ischaemic heart disease, and arrhythmia, although no impact was observed on cardiac arrests or death (39). A comparative study based on US administrative claims data provided real world data showing the increased risk for hospitalisations for heart failure among castrate-resistant prostate cancer patients on abiraterone compared to enzalutamide (HR 2.56, 95% CI 1.32 - 4.94) (40).

### 4.2 De-escalation of ADT

Given that intensification of ADT to improve overall survival for mHSPC patients leads to considerable long-term toxicity, some have advocated for the de-escalation of ADT to minimise the toxicity of treatment while maintaining its efficacy. While there is currently no high-level evidence for intermittent or de-escalated ADT in the setting of combination therapy, and comparison between continuous and intermittent ADT still remains controversial (41, 42), it may be possible to employ de-escalation as a reasonable strategy in certain groups of patients.

#### 4.2.1 Suitable patient groups for potential de-escalation of hormonal treatment

One such group is the exceptional responders to hormonal treatment. Various pivotal trials (42–44) have shown that undetectable PSA levels six to seven months after initiation of ADT is a highly favourable prognostic factor. When comparing the survival benefit that this stratification brings about, it may seem that the benefit surpasses the effect that metastatic volume has on overall survival, allowing for cross-trial differences (42, 45). Given their extraordinary treatment outcomes and prognosis in relation to prostate cancer, the greater risk toward overall survival in these patients seems to tip towards cardiovascular morbidity. In such cases, there may be a role in select patients for de-escalation of ADT to reduce the lifetime exposure of associated adverse cardiovascular effects. This offers an optimal balance towards improving the overall survival of these patients, not to mention quality of life.

Another group of patients would be those with favourable disease factors with respect to prostate cancer. The CHAARTED (43) and GETUG-AFU 15 (46) trials have shown that metachronous, low-volume metastatic prostate cancer portends the best median overall survival (8 years). This is in significant contrast to median overall survival in metachronous, high-volume disease (4.5 years); synchronous, low-volume disease (4.5 years); and synchronous, high-volume disease (3 years). Furthermore, the recent ENZAMET (47) trial found that five-year overall survival for low-volume disease exceeded 80% in both metachronous and synchronous disease, compared to 50% in synchronous, high-volume disease. Overall, given the relatively favourable prognosis of prostate cancer patients with metachronous, low-volume disease, these findings may tip the risk-benefit ratio for these patients in favour of ADT de-escalation, to reduce mortality caused by adverse cardiovascular outcomes.

Thirdly, certain patient factors may give clinicians pause to consider a role for de-escalation of ADT. Examples of this might include patients who are experiencing significant cardiovascular toxicity from ADT, frail elderly patients with multiple life-limiting comorbidities at baseline, and patients who choose to prioritize their quality of life after receiving a diagnosis. Notably, the FinnProstate Study VII (48) found that intermittent ADT improved the quality of life in patients receiving treatment for advanced prostate cancer, although it did not reduce the overall prevalence of adverse effects. Nonetheless, there is currently a lack of robust data in determining patient factors which favour de-escalated or intermittent ADT, and clinicians would need to engage patients in a shared decision-making framework should they wish to offer de-escalation based on patient factors.

Lastly, there is potential for the use of predictive biomarkers in identifying patients who are suited for de-escalated ADT. Swami and colleagues (49) have reported that the presence of SPOP mutations - found in approximately 5% of mHSPC patients - is predictive of a highly favourable response to ADT plus ARPi therapy, though not ADT plus docetaxel therapy. Hearn and colleagues (50) determined that the HSD3B1 variant genotype is a valid and powerful predictor of patients who are likely to exhibit resistance to ADT or more aggressive disease, warranting early escalated therapy and thus reducing their suitability for de-escalation. Exploring mechanisms of AR resistance may also uncover potential biomarkers that may guide avoidance of ARPi due to expected poor response and at the same time, reduce unnecessary exposure to the negative cardiovascular effects of these agents mentioned earlier. The androgen receptor splice variant 7 (AR-V7), a splice variant of the androgen receptor mRNA that leads to truncation of the ligand-binding domain, has been shown to be a biomarker for resistance to androgen receptor pathway targeted agents (51). Other prostate cancer phenotypes which demonstrate a diminished AR pathway signalling or response to AR targeted agents deserve further investigations. AR indifference describes a state in which the cells do not depend on AR activity despite continued AR expression and studies support the deregulation of cyclin D/E, E2F1, RB1, and increased Myc activity mediated hyper-activation of the E2F cell-cycle master regulator as the driver of growth in these AR indifferent cells (52). Furthermore, patients who display minimal residual disease on prostate-specific membrane antigen positron emission tomography (PMSA PET) scans may also be candidates for de-escalated ADT, although PMSA PET imaging has yet to be validated as a tool to identify exceptional responders to treatment.

Currently, there are numerous ongoing trials (53) to evaluate the outcomes of intermittent ADT. A-DREAM is a Phase II trial (ClinicalTrials.gov identifier: NCT05241860) that focuses on exceptional responders to hormonal treatment, whereby mHSPC patients receiving ADT plus ARPi therapy whose PSA levels remain below 0.2 ng/mL after 18 to 24 months will undergo an interruption of treatment. Similarly, the De-Escalate trial (EORTC-2238 GUCG) looks at suspending treatment after 6 to 12 months of ADT plus ARPi therapy in patients who achieve a PSA of 0.2 ng/mL or lower. PREDICT-RT is another Phase III trial (ClinicalTrials.gov identifier: NCT04513717) evaluating the outcomes of less intense hormone and radiation therapy in patients who have high-risk prostate cancer and low gene risk score. The outcomes of these trials will contribute significantly to identifying patients for whom de-escalated or intermittent ADT is a viable option, in order to reduce cardiovascular adverse effects and improve overall survival.

#### 4.2.2 Role of metastasis-directed therapy in ADT de-escalation

The role of metastasis-directed therapy (MDT) to consolidate responses before de-escalation is also worth studying. MDT offers the potential to reduce these toxicities by delaying the progression of metastatic disease and in some situations, eradicating them. Exerting targeted control over these sites of early disease spread also allows the delay of systemic treatment, which in turn limits a patient’s duration of androgen suppression and ADT-related toxicities (43, 54).

There are several approaches to the management of oligometastases. Available modalities include metastasectomy, stereotactic body radiation therapy (SBRT), or a combination of these therapies with or without ADT. However, current studies suffer from small numbers, heterogeneous inclusion criteria, and a lack of standardized outcome measures as well as long-term data. Hence, a universal approach and consensus is still lacking (54, 55).

Among the different strategies, SBRT has the largest body of evidence and several systematic reviews and meta-analyses (SRMA) offer insights into its effect on oligometastatic prostate cancer (OMPC). Ost et al. showed that for patients with metachronous OMPC who underwent salvage MDT (radiotherapy 66%, lymph node dissection 34%), a 3-year progression-free survival (PFS) of 51% could be achieved, although majority (61%) had adjuvant ADT (54). There were more grade 2 complications from lymph node dissection compared to radiotherapy (11% *vs* 8.5%). In another SRMA that included 653 patients from 10 studies with metachronous OMPC (3–5 lesions based on new-generation imaging with PET/CT) treated with SBRT, the 2-year biochemical PFS, radiographic PFS, and ADT-free survival were 33% (95% CI, 11%–55%), 39% (95% CI, 24%–54%), and 52% (95% CI, 41%–62%), respectively. Another SRMA assessing the efficacy of SBRT for OMPC recurrence reviewed data from 23 studies and 1441 treated lesions, showing a PFS and ADT-free survival of 0.413 (95% CI, 0.378–0.477), and 20.1 months (95% CI, 14.5–25.6), respectively. The authors also demonstrated dose-dependent relationship with rates of local control, along with low rates of toxicities (11, 20).

A pooled analysis of the only two prospective randomized trials in MDT (56), namely the STOMP (57) and ORIOLE (58) trials, provided insight into long-term outcomes of MDT and showed a sustained benefit in median PFS with MDT compared to observation (pooled hazard ratio [HR], 0.44; 95% CI, 0.29 to 0.66; P value <.001). High-risk mutations (somatic mutations within ATM, BRCA1, BRCA2, Rb1, or TP53) further risk-stratified these patients, with an improved PFS seen in those without such mutations (PFS 13.4 months *vs* 7.5 months; HR 0.53; 95% CI, 0.25 to 1.11; p = 0.09) (54). Whether conventional imaging or newer molecular imaging such as PSMA is used, the data shows a trend towards clinical benefits of MDT, and ongoing trials will serve to further consolidate the experience with MDT.

### 4.3 Cardiovascular risk assessment and care

Given that a higher prevalence of conventional CVRFs has been reported among cancer survivors (34, 59), cardiovascular risk assessment is, and must be, an important aspect of care for prostate cancer patients. While no dedicated cardiovascular toxicity risk calculators have been developed for patients receiving ADT, the European Society of Cardiology (ESC) has recommended the use of the SCORE2 or SCORE2-OP risk assessment models to perform a baseline cardiovascular risk assessment, as well as estimate 10-year fatal and non-fatal cardiovascular disease risk among patients with no previous cardiovascular disease (60).

In addition to this, the 2022 ESC guidelines (61) support the use of the Heart Failure Association and International Cardio-Oncology Society baseline risk stratification score (62), which was developed for seven classes of potentially cardiotoxic cancer therapies including ADT for prostate cancer (62). The American Heart Association recommends regular monitoring for reversible CVRFs as well as development of the metabolic syndrome every three months (63). In a shared care model involving the oncologist, urologist, cardiologist, and primary care physician, patients undergoing ADT should consider a baseline evaluation which includes (64):

- Vitals: Body mass index, blood pressure.
- History of pre-existing symptoms such as exertional chest pain or dyspnoea, and medical conditions such as diabetes mellitus, hypertension, ischemic heart disease, or stroke.
- Investigations: HbA1c, fasting glucose, lipid panel, with or without electrocardiography.

**Figure 2:**
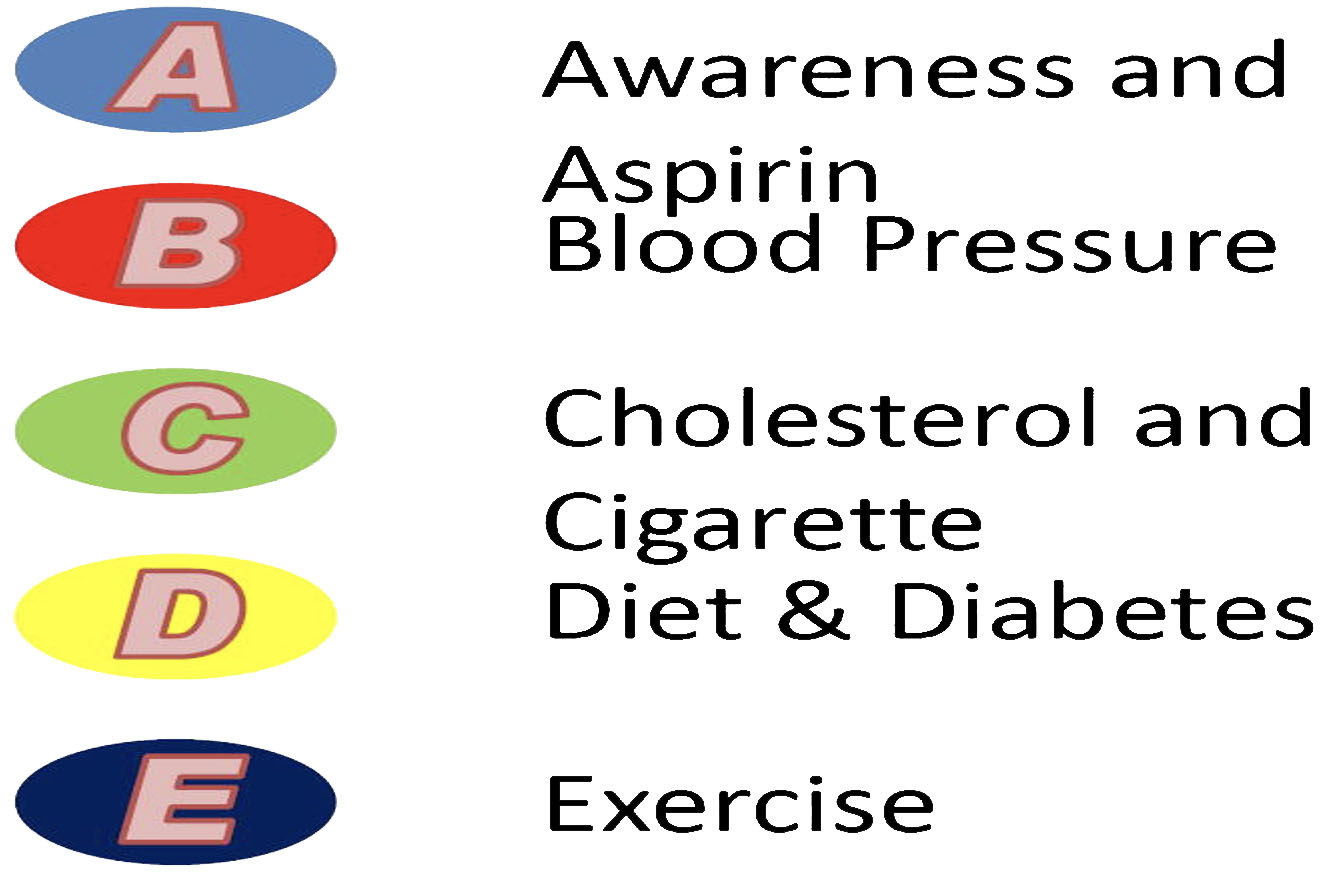
“ABCDE” cardio-oncology framework for prostate cancer patients and management of the CVD risk factors: assessment of risk, antiplatelet/anticoagulant therapy, blood pressure, cholesterol, cigarette/tobacco cessation, diet and weight management, diabetes prevention and treatment, and exercise, as depicted by Bhatia et al. (63).

Bhatia and colleagues (65) have also developed a useful ‘ABCDE’ framework for the reduction of cardiovascular disease in patients with prostate cancer. The algorithm, which Bhatia et al. simplify pictorially in **Figure 2**, consists of the following:

‘A’ refers to increasing Awareness among patients of cardiovascular signs and symptoms, as well as 81mg of Aspirin daily for prevention of cardiovascular events.
‘B’ refers to maintaining a Blood pressure of 140/90mmHg or below.
‘C’ refers to high-intensity statin therapy for pre-existing hyperlipidaemia (Cholesterol control), as well as smoking cessation (Cigarettes).
‘D’ refers to establishing good control of Diabetes mellitus, and cultivating a balanced Diet, which also includes adequate intake of calcium and Vitamin D.
‘E’ refers to Exercise, specifically 150 minutes per week of moderate-intensity physical activity or 75 minutes per week of high-intensity physical activity.

The involvement of a multidisciplinary team is also crucial in optimizing holistic medical care for prostate cancer patients. This includes not only the presence of a cardiologist, but also relevant referrals and consults with endocrinologists and/or family physicians for optimal control of diabetes and hyperlipidaemia (10).

### 4.4 Optimizing patient factors - a focus on exercise

Lastly, patients themselves play an equally important role in reducing their own cardiovascular risk through the undertaking of deliberate, constructive lifestyle modifications. Exercise stimulates a wide range of hormones and cytokines mobilizing the immune system to facilitate repair and rebuild. This reduces systemic inflammation, improves metabolic health, and may even suppress growth and metastasis of prostate cancer. Exercise is increasingly recognized as new medicine improving the quality of life, physical function, mental health and potentially cancer specific survival for men with prostate cancer. In particular, aerobic and resistance exercise has been recommended to prevent and manage cardiovascular disease (10, 65).

Indeed, a systemic review and meta-analysis by Bigaran and colleagues (66) found that compared to usual care, exercise training improved patient outcomes on the 400-m-walk test, diastolic blood pressure, fasting blood glucose, C-reactive protein, whole-body lean mass, appendicular lean mass, whole-body fat mass, whole-body fat percentage, and trunk fat mass.

The general recommendation for unsupervised exercise is to combine aerobic exercise (75 minutes of vigorous to 150 minutes of moderate exercise per week) with resistance training (20–30 minutes x 2–3 times per week). For those who are unable to perform exercise safely or independently, outpatient rehabilitation with supervised training is recommended. For men with bone metastases, the sites of metastatic skeletal lesions need to be evaluated and a tailored exercise program is designed to control loading on these areas (67, 68).

Building on this promising aspect of therapy, several trials are now ongoing to incorporate healthy lifestyle modifications into patient care to reduce cardiovascular morbidity associated with ADT. For example, ProTrio (ClinicalTrials.gov identifier: NCT05054296) is a Phase II trial studying the effectiveness of a structured exercise program and continuous Fitbit monitoring in modifying the risk of metabolic syndrome and cardiovascular disease among prostate cancer patients receiving ADT (69). On a related note, INTERVAL - GAP4 (ClinicalTrials.gov identifier: NCT02730338) is a multicentre, randomized controlled Phase III study aiming to determine if supervised high-intensity aerobic and resistance training increases overall survival compared to self-directed exercise among patients with metastatic castrate-resistant prostate cancer (70).

The results of these studies may provide robust, evidence-based routines for patients to collaborate with care teams and play an active role in the mitigation of their own cardiovascular risk.

## 5 Conclusion

While overall survival among advanced prostate cancer patients is rising, the significant and increasing cardiovascular adversity associated with ADT and intensification of treatment with next-generation hormonal treatment threatens to buck this trend. Active consideration of evidence-based mitigation strategies and close collaboration between multidisciplinary care teams is paramount to control cardiovascular risk, and improve holistic medical outcomes for patients with prostate cancer.

## Data availability statement

The original contributions presented in the study are included in the article/supplementary material. Further inquiries can be directed to the corresponding author.

## Author contributions

KC: Writing – review & editing, Writing – original draft, Visualization, Supervision, Project administration, Conceptualization. TW: Writing – review & editing, Writing – original draft, Visualization, Project administration. YT: Writing – review & editing. KT: Writing – review & editing. WT: Writing – review & editing. JC: Writing – review & editing. HH: Writing – review & editing. CC: Writing – review & editing. JT: Writing – review & editing. PC: Writing – review & editing. HW: Writing – review & editing. MS: Writing – review & editing. RK: Writing – review & editing. YL: Writing – review & editing. CN: Writing – review & editing. JT: Writing – review & editing. JY: Writing – review & editing, Supervision.
